# Modulation of depression-related behaviors by adiponectin AdipoR1 receptors in 5-HT neurons

**DOI:** 10.1038/s41380-020-0649-0

**Published:** 2020-01-24

**Authors:** Chen Li, Fantao Meng, Jacob C. Garza, Jing Liu, Yun Lei, Sergei A. Kirov, Ming Guo, Xin-Yun Lu

**Affiliations:** 1 Institute for Metabolic & Neuropsychiatric Disorders, Binzhou Medical University Hospital, Binzhou, Shandong China; 2 Department of Neuroscience & Regenerative Medicine, Medical College of Georgia at Augusta University, Augusta, GA USA; 3 Present Address: Center for Genomic Medicine and Department of Psychiatry, Massachusetts General Hospital, Harvard Medical School, Boston, MA USA

**Keywords:** Depression, Neuroscience

## Abstract

The adipocyte-derived hormone adiponectin has a broad spectrum of functions beyond metabolic control. We previously reported that adiponectin acts in the brain to regulate depression-related behaviors. However, its underlying neural substrates have not been identified. Here we show that adiponectin receptor 1 (AdipoR1) is expressed in the dorsal raphe nucleus (DRN) and colocalized with tryptophan hydroxylase 2 (TPH2), a marker of serotonin (5-HT) neurons. Selective deletion of AdipoR1 in 5-HT neurons induced anhedonia in male mice, as indicated by reduced female urine sniffing time and saccharin preference, and behavioral despair in female mice and enhanced stress-induced decrease in sucrose preference in both sexes. The expression levels of TPH2 were downregulated with a concurrent reduction of 5-HT-immunoreactivity in the DRN and its two major projection regions, the hippocampus and medial prefrontal cortex (mPFC), in male but not female mice lacking AdipoR1 in 5-HT neurons. In addition, serotonin transporter (SERT) expression was upregulated in both DRN projection fields of male mice but only in the mPFC of female mice. These changes presumably lead to decreased 5-HT synthesis and/or increased 5-HT reuptake, thereby reducing 5-HT transmission. The augmented behavioral responses to the selective serotonin reuptake inhibitor fluoxetine but not desipramine, a selective norepinephrine reuptake inhibitor, observed in conditional knockout male mice supports deficient 5-HT transmission underlying depression-related phenotypes. Our results indicate that adiponectin acts on 5-HT neurons through AdipoR1 receptors to regulate depression-related behaviors in a sex-dependent manner.

## Introduction

Adiponectin is a hormone secreted almost exclusively by adipocytes [1, 2]. In plasma, adiponectin exists as either a full-length protein or a globular fragment generated by proteolytic cleavage of the full-length protein [3, 4]. Full-length adiponectin is the predominant circulating form and can be found as a trimer, hexamer, or high-molecular weight oligomer [5, 6]. Multiple lines of evidence indicate that adiponectin enters the brain from circulation. Trimeric and hexameric forms of adiponectin can be found in the cerebrospinal fluid (CSF) of humans and animals [7–10] and intravenous injection of adiponectin increases CSF adiponectin levels [10, 11]. Furthermore, adiponectin can be detected in the CSF of homozygous adiponectin-deficient mice after intravenous injection of full-length adiponectin, indicating that adiponectin can cross the blood–brain barrier [9, 12].

Low plasma adiponectin levels are commonly associated with insulin resistance in obesity and type 2 diabetes [13, 14], which can increase the risk of depression and anxiety [15, 16]. Some clinical studies indicate a negative correlation between depression severity and circulating adiponectin [17–20]. The association between hypoadiponectinemia and depression remains significant even after controlling for metabolic status or excluding obesity and diabetes [17–19], suggesting that low adiponectin levels can be an independent risk factor for depression. Our preclinical studies have found that adiponectin haploinsufficiency or immunoneutralization of adiponectin in the brain increases the susceptibility to depression-related behaviors [21]. By contrast, intracerebroventricular administration of exogenous adiponectin produces antidepressant-like behavioral effects in normal and diet-induced obese mice [21]. These results suggest that adiponectin regulates depression-related behaviors through a central mechanism.

Adiponectin exerts its effects via two distinct receptors, AdipoR1 and AdipoR2. Both receptors contain seven transmembrane domains but, unlike G-protein coupled receptors, have an intracellular amino terminus and an extracellular carboxyl terminus [22]. AdipoR1 and AdipoR2 have distinct distribution patterns and bind to various forms of adiponectin with different affinity [22]. While AdipoR1 is widely expressed in the brain, AdipoR2 is restricted to only a few brain regions [9, 21]. We have shown that AdipoR1 and AdipoR2 signaling in the hippocampus and ventral tegmental area are involved in the regulation of anxiety-related behavior and fear memories in a mouse model of post-traumatic disorder [23, 24]. However, the specific roles of AdipoR1 and AdipoR2 in depression-related behaviors and the underlying neural substrates remain to be elucidated.

The serotonin (5-HT) system has been implicated in the pathophysiology and treatment of depression [25, 26]. A central role of 5-HT in depression is supported by therapeutic actions of augmenting 5-HT function, more specifically, increasing 5-HT levels in the synaptic cleft [27, 28]. Most of antidepressants, especially selective serotonin reuptake inhibitors (SSRIs), increase synaptic 5-HT activity [29]. On the other hand, several lines of evidence suggest the presence of 5-HT deficiency in depression [30]. Acute depletion of tryptophan, an essential amino acid precursor of 5-HT, induces depressive relapse in remitted patients with major depressive disorder by lowering serotonin synthesis [31, 32]. Treatment with the vesicular monoamine transporter inhibitor reserpine depletes monoamines, resulting in depression [33], which is often cited as the earliest evidence in support of the monoamine hypothesis of depression. Moreover, low CSF concentrations of 5-hydroxyindoleacetic acid (5-HIAA), the metabolite of 5-HT, were found in depressed patients [34], and mutations of genes encoding key components of the 5-HT system have been associated with affective disorders and stress sensitivity [35–37]. However, the effects of brain 5-HT deficiency on stress susceptibility and the development of depression-related behaviors are not well understood.

In this study, we investigated the anatomical basis of functional interactions between adiponectin signaling and the 5-HT system. We generated AdipoR1 conditional knockout mice and determined the impact of loss of AdipoR1 specifically in 5-HT neurons on depression- and anxiety-related behaviors, expression of key components of the 5-HT system and antidepressant responses in both male and female mice.

## Materials and Methods

### Animals

Wild-type (WT) C57BL/6J mice (Stock No. 000664) were purchased from Jackson Laboratory (Bar Harbor, ME, USA). Ai14 mice that express robust tdTomato fluorescence following Cre-mediated recombination (Stock No. 007908) and mice hemizygous for the ePet-Cre transgene (Stock No. 012712) that were backcrossed to a C57BL/6J background were obtained from Jackson Laboratory. To confirm the specificity of ePet-Cre-mediated recombination in serotonin (5-HT) synthesis neurons, ePet-Cre mice were crossed with the Ai14 tdTomato reporter line to obtain ePet-Cre, Ai14 mice with tdTomato fluorescence in Cre-expressing cells, which was used to determine the colocalization of tdTomato with 5-HT neuron-specific markers. To generate conditional knockout mice lacking AdipoR1 in 5-HT neurons, AdipoR1^flox/flox^ (AdipoR1^f/f^) mice, in which exon 2 is floxed [23], were crossed with ePet-Cre mice. The AdipoR1^flox/+^, ePet-Cre offspring were backcrossed with AdipoR1^f/f^ mice to generate AdipoR1^f/f^, ePet-Cre (AdipoR1^f/f;ePet-Cre^), and AdipoR1^f/f^ littermate controls. The PCR primers used for genotyping were as follows: ePet-Cre, forward-5′-GCG GTCTGGCAGTAAAAACTATC-3′, reverse-5′-GTGAAACAGCATTGCTGTCACTT-3′, and AdipoR1 WT and flox, forward-5′-CCCTGGGGATAGTTCTGGAT-3′, reverse-5′-TTACTCACTGGGCCCTGCTTG-3′. All mice were housed in groups of five per cage under a 12-h light/dark cycle (lights on at 7:00 a.m.) with *ad libitum* access to water and standard food pellets. Both male and female mice at an age between 8 and 15 weeks were used and all animal procedures were approved by the Institutional Animal Care and Use Committee of Binzhou Medical University Hospital and Augusta University.

### Drugs

Fluoxetine and desipramine (Sigma-Aldrich, ST. Louis, MO, USA) were dissolved in saline (0.9% NaCl w/v) to a final concentration of 1 mg mL^−1^ for intraperitoneally (i.p.) injection with a dose of 5 or 10 mg kg^−1^.

### Quantitative real-time PCR analysis

Mice were decapitated and the brains were removed rapidly. The medial prefrontal cortex (mPFC) and hippocampus were immediately dissected on ice. The DRN was dissected from five to six 100-μm-thick coronal midbrain slices (from −4.36 to −4.96 mm from bregma) on ice according to the mouse brain atlas [38]. Total RNA was extracted from each tissue sample to generate cDNA using a protocol reported elsewhere [39, 40]. First, total RNA was treated with 4× gDNA wiper mix at 42 °C for 2 min to remove genomic DNA contamination, then reversely transcribed into cDNAs with 5× HiScript II QRT SuperMix that was added to the reaction mixture and incubated at 25 °C for 10 min followed by 50 °C for 30 min and 85 °C for 5 min. The resulting cDNA was used for real-time PCR detection using the StepOnePlus real-time PCR system (Applied Biosystems, Waltham, MA, USA). The condition for PCR was 95 °C for 5 min, followed by 40 cycles of 95 °C for 10 s and 60 °C for 30 s. The primer sequences used to amplify each desired product were as follows: mouse AdipoR1 exon 2 [23], forward-5′-CCCGTATCCACCAGACACCGG-3′, reverse-5′-GGCAATGGGGCTCCTTCTGG-3′; mouse β-tubulin [39], forward-5′-AGCAACATGAATGACCTGGTG-3′, reverse-5′-GCTTTCCCTAACCTGCTTGG-3′. The housekeeping gene β-tubulin was used as a reference gene for normalization of gene expression. The 2^−ΔΔCT^ method, i.e., delta-delta-ct analysis, was used as a relative quantification [40, 41].

### In situ hybridization

Radioactive in situ hybridization for detecting AdipoR1 and AdipoR2 gene expression patterns were performed as previously described [21, 24]. Briefly, the brain tissue (fresh frozen) was cut at a thickness of 20 μm, mounted onto poly-lysine coated slides and sequentially fixed in 4% paraformaldehyde for 1 h followed by a rinse in 2× saline sodium citrate (SSC) buffer (300 mM NaCl, 30 mM Na citrate, pH 7.2), acetylated in 0.1 M triethanolamine (pH 8.0) with 0.25% (vol/vol) acetic anhydride for 10 min and dehydrated through a graded series of alcohol (50–100%). AdipoR1 and AdipoR2 cDNA fragments were amplified by RT-PCR using the primers as follows: AdipoR1 (721 bp), forward: 5′-CCGCTCGAGGATCGGGCGCCCCTC-3′ and reverse: 5′-CGGGATCCCGAAGACCACCTTC-3′; and AdipoR2 (912 bp), forward: 5′-CCGCTCGAGTGTAACATGGGCCTCG-3′ and reverse: 5′-CGGGATCCCCAAAAGTGTGC-3′. The cDNA fragments were cloned into pBluescript II SK+ vector (Stratagene, San Diego, CA, USA). To generate radiolabeled cRNA probes, the plasmids were linearized and labeled with S^35^-UTP and S^35^-CTP (PerkinElmer, Waltham, MA, USA) using the standard transcription system. The brain tissue sections were incubated with 70 μl of radioactive S^35^-labeled AdipoR1 or AdipoR2 cRNA probes at 55 °C overnight, rinsed in 2 × SSC and incubated in RNase A buffer (200 mg/ml) for 1 h at 37 °C followed by a series of washes with increasing stringency (2×, 1×, 0.5× SSC). Finally, the sections were placed in 0.1 × SSC at 70 °C for 1 h, rinsed in distilled water, dehydrated in a graded series of alcohol, and exposed to X-ray film.

### Combined in situ hybridization and immunohistochemistry

To examine whether AdipoR1 mRNA is colocalized with 5-HT neurons in the DRN, we performed in situ hybridization to detect AdipoR1 mRNA in combination with immunohistochemistry to detect TPH2, a marker of 5-HT neurons [24]. Mouse AdipoR1 cDNA fragments were amplified by RT-PCR using the primers as follows: forward: 5′-GGGATGTTCTTCCTGGGCGCGGTGCTCTGCCT-3′ and reverse: 5′-ACTCCTGCTCTTGTCTGCCGGTGCTTGGGAGTGG-3′. These PCR amplified fragments were cloned into pBluescriptSKII+ vector. AdipoR1 cRNA probes were labeled with digoxigenin-11-UTP (Sigma-Aldrich, ST. Louis, MO, USA) using standard transcription methods. The brain sections were hybridized with a 1:1 mixture of the labeled cRNA probes at 55 °C overnight and subsequently rinsed in 2 × SSC, treated with RNase A (200 μg/ml) for 1 h at 37 °C, and washed in 2 × , 1 × , 0.5 × , and 0.1 × SSC (for 5 min each), followed by a final rinse in 0.1 × SSC at 65 °C for 1 h. Then, brain sections were treated with 2% hydrogen peroxide in 0.05 M phosphate-buffered saline (PBS) for 30 min followed by incubation with a blocking solution (PerkinElmer) for 1 h and a sheep anti-digoxigenin antibody conjugated to horseradish peroxidase (HRP; Roche, Basel, Switzerland) overnight in a humidified chamber. The sections were rinsed in PBS and then incubated with anti-TPH2 antibody (1:400, ab184505, Abcam, Cambridge, UK) overnight in a humidified chamber, followed by incubation with Alexa Fluor 488 goat anti-rabbit IgG secondary antibody (1:400; Molecular Probes, Eugene, OR, USA) for 4 h and the TSA Plus cyanine 5 tyramide amplification reagent (PerkinElmer, Waltham, MA) for 20 min. After rinsing, the slides were coverslipped immediately with the ProLong Gold antifade reagent (Invitrogen, Carlsbad, California, USA). Signal specificity was ensured either by hybridization with sense-strand probes or pretreatment of brain sections with RNase A (200 μg/ml at 37 °C for 1 h). The colocalization of AdipoR1 mRNA and TPH2 protein was evaluated in the raphe nuclei using a fluorescence microscope. Neurons from three brain sections containing the DRN of each animal were counted.

### Western blot assay

The dissected brain tissue samples (mPFC, hippocampus and DRN) were homogenized in a lysis buffer containing 50 mM Tris-HCl buffer, 150 mM NaCl, 1% Triton X-100, 1% sodium deoxycholate, 0.1% sodium dodecyl sulfate (SDS), phenylmethylsulfonyl fluoride, and PhosSTOP Phosphatase Inhibitor Cocktail (Roche Applied Science, Penzberg, Germany). The denatured protein was separated on a SDS–polyacrylamide gel electrophoresis and transferred to polyvinylidene fluoride membrane. The membrane was blocked in a solution of tris-buffered saline with 1% dried milk and 0.1% tween 20, and then incubated with the following primary antibodies diluted in a blocking solution: anti-TPH2 antibody (1:1000; ab184505, Abcam, Cambridge, UK), anti-SERT antibody (1:1000; ab172884, Abcam, Cambridge, UK), anti-β-actin antibody (1:1000, #4970, Cell Signaling Technology, Danvers, MA, USA). After washing, the membrane was incubated with IRDye 680LT donkey anti-rabbit IgG secondary antibodies (1:5000; 926-68023, Li-COR Biosciences, Lincoln, NE, USA). The fluorescence was visualized and analyzed using an Odyssey Infrared Imaging System (Li-COR Biosciences, Lincoln, NE, USA).

### Immunofluorescence histochemistry and semi-quantitative assessment

Mice were transcardially perfused with 4% paraformaldehyde. The brains were removed, postfixed overnight and then transferred to 30% sucrose and cut into 40-μm coronal sections. Immunohistochemistry was performed to detect TPH2, SERT, and 5-HT in the raphe nuclei and 5-HT in the PFC and hippocampus. Briefly, brain sections were rinsed three times in PBS, and incubated in a blocking buffer (1% bovine serum albumin, 3% goat serum, 0.3% Triton X-100 in PBS) for 1 h. The sections were then incubated with anti-TPH2 (1:400, ab184505, Abcam, Cambridge, UK), anti-SERT (1:200, ab130130, Abcam), and anti-5-HT (1:400; S5545, Sigma, Saint Louis, USA) overnight, respectively. After washing in PBS, sections were incubated for 4 h with Alexa Fluor 488 goat anti-rabbit IgG secondary antibody (1:400; Molecular Probes, Eugene, OR, USA) at room temperature. Finally, the sections were washed in PBS, mounted onto poly-lysine-coated glass slides, coverslipped with fluorescence mounting medium and visualized using an Olympus confocal microscope (Olympus, Shinjuku, Tokyo, Japan). We analyzed immunofluorescence intensity in the DRN, mPFC and hippocampus using ImageJ (http://imagej.nih.gov/ij/) and counted the number of cells immunopositive for SERT, TPH2, and 5-HT in 3–5 sections (DRN and mPFC) or 7–8 sections (hippocampus) from each brain spaced every 120 μm from approximately −4.36 to −4.96 mm for the DRN, from 1.34 to 1.98 mm for the mPFC and every 240 μm from −1.34 to −3.64 mm for the hippocampus relative to bregma. The immunopositive cells were counted manually by experimenters who were blind to the genotypes. Images of immunofluorescence staining were converted to grayscale and the signal intensity of immuolabeling in the DRN, mPFC, and hippocampus was quantified as integrated density using the ImageJ software. The mean gray values were calculated as: [integrated density − (measured area × mean background signal)]/measured area. The background signals were measured in areas without signal. The densities were averaged across the sections from each brain and expressed as a percentage relative to the control group mean.

### Behavioral and stress procedures

All behavioral procedures were performed in the late light cycle except sucrose and saccharin preference tests that were measured in the first 2 h in the dark cycle. The behaviors of each mouse were scored by experimenters who were blind to the genotypes or treatment conditions. Mice were subjected to multiple behavioral tests spaced at least 3 days apart to decrease possible carry-over effects from prior tests.

### Chronic unpredictable stress (CUS)

Mice were subjected to two different stressors daily at different times of the day and housed singly for five consecutive days [39]. The stressors included 2-h restraint, 15-min tail pinch, 24-h constant light, 24-h wet bedding, and 45° cage tilt, 10-min inescapable foot shocks, 30-min elevated platform, and social isolation. Stress exposure was conducted in a procedure room. Control mice were handled daily.

### Female urine sniffing test

This test was used to assess sex-related reward-seeking behavior based upon interest of male rodents in pheromonal odors from estrus female urine [39, 42]. Female mice were maintained in a separate room, and the estrous cycle was assessed by microscopic evaluation of vaginal smears as previously described [43]. Urine from estrus female mice was collected and stored on ice before use. One hour before the test, mice were habituated to a sterile cotton-tipped applicator inserted into their home cage. For the test, mice were transferred to a dimly-lit room (about 3 lux lighting). The test had three phases: (1) one exposure (3 min) to the cotton tip dipped in sterile water; (2) a 45-min interval; and (3) one exposure (3 min) to a cotton tip applicator infused with fresh urine (80 μL) collected from female mice of the same strain in estrus. Sniffing time was recorded during exposure to water and female urine.

### Saccharin/sucrose preference test

Mice were habituated to drinking water from two bottles for 1 week before testing. Mice were provided with a free choice between a bottle containing 1% sucrose or 0.01% saccharin solution and a bottle of plain water. Water and saccharin/sucrose intake was measured during the first 2 h after lights off in the dark cycle. The preference for sucrose or saccharin solution was calculated by dividing the mass of sucrose or saccharin solution consumed by the total mass of fluid intake. For mice subjected to 5 days of chronic unpredictable stress, sucrose preference test was performed 24 h after the last stressor.

### Forced swim test

Mice were placed in a clear Plexiglas cylinder (25 cm high; 10 cm in diameter) filled with 24 °C water to a depth of 15 cm. A charge-coupled device (CCD) camera positioned directly above the cylinder was used to record the behavior of each mouse for 6 min. The duration of immobility in the last 4 min was measured. Immobility was defined as no movement except those caused by respiration [40].

### Locomotor activity

The locomotor activity was measured in an open-field box (40 × 40 × 40 cm). Mice were placed in the open-field area and allowed to freely explore for 30 min [39, 40]. A CCD camera was mounted above the open box for recording locomotor activity. The total distance traveled was measured in 2-min bins using Any-maze software (Stoelting, Wood Dale, IL, USA).

### Elevated plus-maze test

The elevated plus-maze test is based on the natural conflict between the animal’s drive to explore a new environment and the tendency to avoid a potentially dangerous [44]. The elevated plus-maze consisted of four arms (35-cm long and 5-cm wide) arranged in the shape of a ‘plus’ sign and elevated to a height of 70 cm from the floor. Two arms have no side or end walls (open arms) and the other two arms have side walls and end walls but are open on top (closed arms). The open and closed arms intersect, having a central 5 × 5-cm square platform giving access to all arms. As described previously [24, 40], mice were placed in the central square facing the corner between a closed arm and an open arm and allowed to explore the elevated plus-maze for 5 min. Their activity was videotaped. The numbers of entries made into each arm and the time spent on the open and closed arms were measured. The approach-avoidance performance was assessed by calculating the percentage of open arm entries (entries into the open arms/total entries into all arms) and percentage of time spent in the open arms (time spent in open arms/total time spent in all arms).

### Open-field test

The apparatus consisted of a 60 × 60 cm open arena with 40 cm high walls. The entire test arena was adjusted to even illumination. Mice were placed in the center of the arena, and their activity was recorded for 5 min. For analysis, the open-field arena was divided into nine equal squares by a 3 × 3 grid. The center square was defined as the central zone, in which the animal’s activity is usually regarded as a measure of anxiety. The time mice spent in the central zone and the total distance traveled in the open arena were quantified using Any-maze software (Stoelting, Wood Dale, IL, USA). The overall motor activity during the open-field test was assessed as the total distance traveled.

### Statistical analyses

Shapiro–Wilk test and F test were used to test the normality and equal variance assumptions, respectively. For normally distributed data, two-tailed *t* tests were used to assess differences between two experimental groups with equal variance. For a two-sample comparison of means with unequal variances, two-tailed *t* tests with Welch’s correction were used. For non-normally distributed data, Mann–Whitney U tests were performed to compare two groups. For locomotor activity, forced swim test, and female urine sniffing test, two-way repeated-measures ANOVAs or two-way ANOVAs followed by Bonferroni tests were used. *P* < 0.05 was considered statistically significant. All data were presented as mean ± standard error (s.e.m.).

## Results

### Expression of AdipoR1 mRNA in dorsal raphe 5-HT neurons

**Fig. 1:**
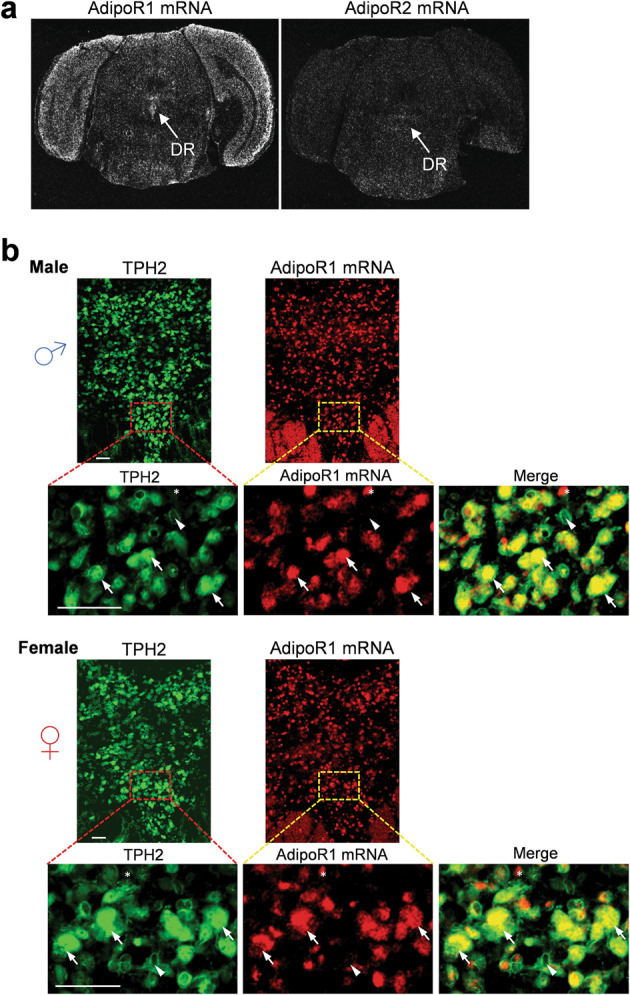
Expression of AdipoR1 and AdipoR2 in the dorsal raphe nucleus. **a** In situ hybridization showing expression patterns of AdipoR1 and AdipoR2 mRNA in the dorsal raphe nucleus. **b** Colocalization of AdipoR1 mRNA and TPH2 protein in the dorsal raphe nucleus on male (upper panel) and female (lower panel) mice. Arrows indicating neurons that contain both AdipoR1 mRNA and TPH2; arrowhead showing neurons that contain only TPH2; asterisk indicating neurons that express only AdipoR1 mRNA only. Scale bar = 50 µm.

Expression of AdipoR1 and AdipoR2 mRNA in the raphe nuclei was examined using in situ hybridization with radioactive cRNA probes. While AdipoR1 mRNA was highly expressed in the DRN, AdipoR2 mRNA was present in very low abundance (Fig. 1a). The degree of colocalization of AdipoR1 and 5-HT neurons in the DRN was assessed using the combined nonradioactive in situ hybridization and immunohistochemical labeling. We found that the vast majority of 5-HT neurons immunoreactive for TPH2 expressed AdipoR1 mRNA in both male and female mice (Fig. 1b; male mice, 97.40% ± 1.33%, total 1289 neurons counted from six brain sections; female mice, 98.80% ± 0.40%, total 1356 neurons counted from six brain sections). This observation provides a neuroanatomical basis for direct action of adiponectin on dorsal raphe 5-HT neurons.

### Generation of mice lacking AdipoR1 specifically in 5-HT neurons

**Fig. 2:**
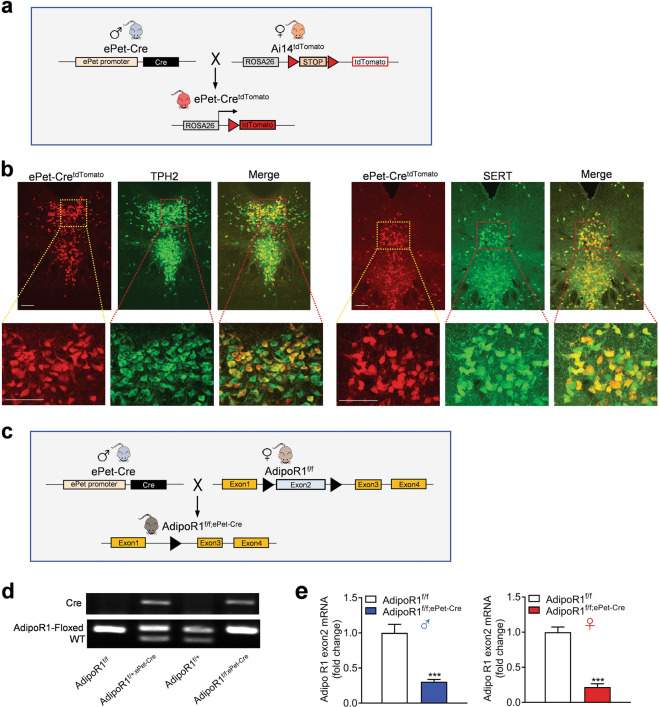
Generation of mice lacking AdipoR1 specifically in 5-HT neurons. **a** Schematic diagram of the strategy used for generation of ePet-Cre^tdTomato^ reporter mice. **b** (Left panel) Representative coronal sections showing colocalization of tdTomato (red) and tryptophan hydroxylase 2 (TPH2, green) in the dorsal raphe nucleus. (Right panel) Representative coronal sections showing colocalization of tdTomato (red) and serotonin transporter (SERT, green). Scale bar = 100 μm. **c** Schematic diagram of generation of mice with selective deletion of AdipoR1 in 5-HT neurons. **d** PCR results of genotyping. **e** Real-time PCR analysis showing Cre-mediated deletion of exon 2 of AdipoR1 in the dorsal raphe nucleus of male and female AdipoR1^f/f;ePet-Cre^ mice. Male: AdipoR1^f/f^: *n* = 7; AdipoR1^f/f;ePet-Cre^: *n* = 6, Female: AdipoR1^f/f^: *n* = 6; AdipoR1^f/f;ePet-Cre^: *n* = 6. ***P < 0.001 compared with AdipoR1^f/f^ littermate controls.

To determine the role of AdipoR1 specifically in 5-HT neurons, we generated 5-HT neuron-specific AdipoR1 knockout mice by crossing homozygous AdipoR1^f/f^ mice with ePet-Cre mice [45]. AdipoR1^f/f^ mice have loxP sites flanking exon 2 of the AdipoR1 gene [23]. The Pet-1 gene is exclusively expressed in 5-HT neurons [46]. In ePet-Cre mice, Cre recombinase was placed under control of the Pet-1 promoter to direct Cre expression to 5-HT neurons [45]. The specificity of ePet-Cre-mediated recombination in 5-HT neurons was confirmed by crossing ePet-Cre mice with the tdTomato reporter line (Ai14) (Fig. 2a), showing that the expression of tdTomato fluorescence was colocalized with the two specific markers for 5-HT neurons, TPH2 (91.20% ± 0.58% tdTomato-labeled neurons positive for TPH2; total 1577 neurons counted from three mice) and SERT (92.20% ± 0.94% tdTomato-labeled neurons positive for SERT; total 1477 neurons counted from three mice) (Fig. 2b). The male AdipoR1^f/+;ePet-Cre^ offspring that resulted from a mating between AdipoR1^f/f^ and ePet-Cre mice were backcrossed with female AdipoR1^f/f^ to generate AdipoR1^f/f;ePet-Cre^ mice and AdipoR1^f/f^ littermate controls for the experiments (Fig. 2c, d). Real-time quantitative PCR revealed the deletion of AdipoR1 exon 2 in the DRN of both male and female AdipoR1^f/f;ePet-Cre^ mice (Fig. 2e, male: *t* test with Welch’s correction, *P* < 0.001; female: *t*_(10)_ = 8.985, *P* < 0.001).

### 5-HT neuron-specific AdipoR1 knockout leads to differential behavioral phenotypes in male and female mice

**Fig. 3:**
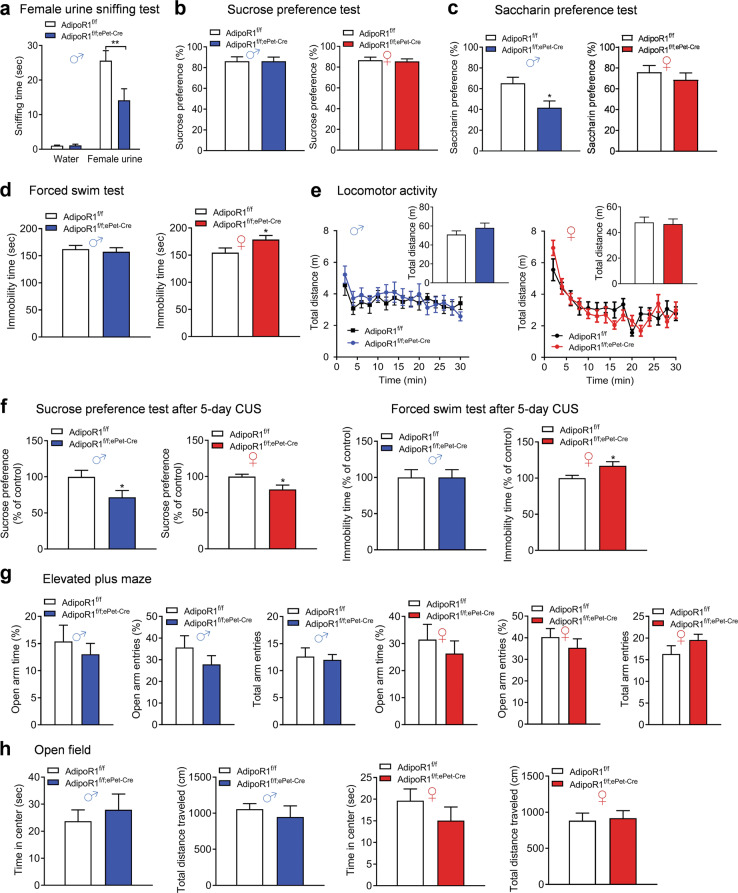
Selective deletion of AdipoR1 in 5-HT neurons induces depression-related behaviors. **a** Female urine sniffing test, AdipoR1^f/f^, *n* = 12; AdipoR1^f/f;ePet-Cre^, *n* = 11. **b** Sucrose preference test. Left, male mice, AdipoR1^f/f^, *n* = 12; AdipoR1^f/f;ePet-Cre^, *n* = 11. Right, female mice, AdipoR1^f/f^, *n* = 11; AdipoR1^f/f;ePet-Cre^, *n* = 14. **c** Saccharin preference test. Left, male mice, AdipoR1^f/f^ *n* = 12; AdipoR1^f/f;ePet-Cre^, *n* = 11. Right, female mice, AdipoR1^f/f^, *n* = 11; AdipoR1^f/f;ePet-Cre^, *n* = 14. **d** Forced swim test. Left, male mice, AdipoR1^f/f^, *n* = 12; AdipoR1^f/f;ePet-Cre^, *n* = 10. Right, female mice, AdipoR1^f/f^, *n* = 11; AdipoR1^f/f;ePet-Cre^, *n* = 14. **e** Locomotor activity. The time course of locomotor activity of male (left) and female (right) mice. The inserts indicating the total distance traveled during the 30-min test. Male mice, AdipoR1^f/f^, *n* = 12; AdipoR1^f/f;ePet-Cre^, *n* = 11. Female mice, AdipoR1^f/f^, *n* = 11; AdipoR1^f/f;ePet-Cre^, *n* = 14. **f** (Left and middle-left) Sucrose preference test performed on mice after 5-day CUS. Male mice: AdipoR1^f/f^, *n* = 8; AdipoR1^f/f;ePet-Cre^, *n* = 8. Female mice: AdipoR1^f/f^, *n* = 11; AdipoR1^f/f;ePet-Cre^, *n* = 12. (Middle-right and right) Forced swim test. Male mice: AdipoR1^f/f^, *n* = 8; AdipoR1^f/f;ePet-Cre^, *n* = 8. Female mice: AdipoR1^f/f^, *n* = 11; AdipoR1^f/f;ePet-Cre^, *n* = 12. **g** Elevated plus-maze test performed on male (left panel) and female (right panel) mice. Male mice: AdipoR1^f/f^, *n* = 10; AdipoR1^f/f;ePet-Cre^, *n* = 9. Female mice: AdipoR1^f/f^, *n* = 9; AdipoR1^f/f;ePet-Cre^, *n* = 9. **h** Open-field test performed on male (left panel) and female (right panel) mice. Male mice: AdipoR1^f/f^, *n* = 10; AdipoR1^f/f;ePet-Cre^, *n* = 9. Female mice: AdipoR1^f/f^, *n* = 10; AdipoR1^f/f;ePet-Cre^, *n* = 8. *P < 0.05, **P < 0.01 compared with AdipoR1^f/f^ littermate controls.

Sex differences in adiponectin have been reported in both humans and mice [47–49], we therefore assessed the consequences of deletion of AdipoR1 in 5-HT neurons on depression- and anxiety-related behaviors in both male and female mice. Dorsal raphe 5-HT neurons can be rapidly activated by various rewards, including sucrose, food, and sex [50, 51]. First, we determined whether AdipoR1 signaling in 5-HT neurons affects sex-related reward-seeking behavior in male mice using the female urine sniffing test, which is based upon interest of male rodents in pheromonal odors from estrus female urine [42]. We found that male AdipoR1^f/f;ePet-Cre^ mice spent less time sniffing female urine (*P* = 0.004), but showed no difference in water sniffing time as compared with AdipoR1^f/f^ mice (*P* = 0.999), indicating an anhedonic-like phenotype (Fig. 3a; genotype, *F*_(1,42)_ = 6.710, *P* = 0.013; sniffing object, *F*_(1,42)_ = 73.450, *P* < 0.001; genotype × sniffing object, *F*_(1,42)_ = 6.870, *P* = 0.012). Anhedonia can be measured by sucrose preference in both male and female mice [39, 52, 53]. Under basal conditions, neither male nor female AdipoR1^f/f;ePet-Cre^ mice showed significant difference in preference for 1% sucrose solution as compared with their relative control AdipoR1^f/f^ mice (Fig. 3b, male: Mann–Whitney test, *P* = 0.786; female: *t*_(23)_ = 0.298, *P* = 0.769). However, when tested using 0.01% saccharin solution, male AdipoR1^f/f;ePet-Cre^ mice exhibited a reduced preference (Fig. 3c, male: *t*_(21)_ = 2.710, *P* = 0.013; female: Mann–Whitney test, *P* *=* 0.345). Female but not male AdipoR1^f/f;ePet-Cre^ mice exhibited increased behavioral despair, as indicated by increased immobility in the forced swim test (Fig. 3d, male: *t*_(20)_ = 0.483, *P* = 0.634; female: *t*_(23)_ = 2.130, *P* = 0.044). No genotype difference in baseline locomotion was observed in either sex (Fig. 3e, male: genotype, *F*_(1,21)_ = 0.205, *P* = 0.655; time, *F*_(14,294)_ = 3.020, *P* < 0.001; genotype × time, *F*_(14,294)_ = 0.798, *P* = 0.671; total distance: *t*_(21)_ = 1.090, *P* = 0.288); female: genotype, *F*_(1,23)_ = 0.048, *P* = 0.828; time: *F*_(14,322)_ = 18.810, *P* < 0.001; genotype × time, *F*_(14,322)_ = 2.239, *P* = 0.007; total distance: *t*_(23)_ = 0.220, *P* = 0.828). To determine whether loss of AdipoR1 in 5-HT neurons increases susceptibility to stress, male and female mice were exposed to 5 days of CUS. Sucrose preference was significantly reduced in both male and female AdipoR1^f/f;ePet-Cre^ mice exposed to 5 days of CUS when compared with AdipoR1^f/f^ control mice (Fig. 3f, male: *t*_(14)_ = 2.175, *P* *=* 0.047; female: *t* test with Welch’s correction*, P* = 0.020), while increased immobility in the forced swim test was only seen in female but not male mice (Fig. 3f, male: Mann–Whitney test, *P* = 0.999; female: *t*_(21)_ = 2.404, *P* = 0.025). These results indicate that loss of AdipoR1 in 5-HT neurons results in differential changes in depression-related behaviors in male and female mice.

Although controversial, studies have shown that 5-HT neurons in the raphe nuclei modulate anxiety behaviors [54–57]. To determine whether AdipoR1 in 5-HT neurons regulate anxiety-related behaviors, we employed the approach-avoidance conflict tests. Avoidance of potential threats and dangerous situations at the cost of approach opportunities is a hallmark feature of anxious disorders. Male and female AdipoR1^f/f;ePet-Cre^ and AdipoR1^f/f^ mice were assessed using the elevated plus-maze test. The percentage of open arm time, the percentage of open arm entries, total arm entries showed no genotype difference in both male and female mice (Fig. 3g, male: percentage of open arm time, *t*_(17)_ = 0.673, *P* = 0.532; percentage of open arm entries, *t*_(17)_ = 1.414, *P* = 0.270; total arm entries, *t*_(17)_ = 0.310, *P* = 0.761; female: percentage of open arm time, *t*_(16)_ = 0.707, *P* *=* 0.490; percentage of open arm entries, *t*_(16)_ = 0.865, *P* *=* 0.400; total arm entries, *t*_(16)_ = 1.406, *P* = 0.179). In the open-field test, neither male nor female mice showed significant genotype difference in time spent in the central zone and total distance traveled (Fig. 3h; male mice: time in the central zone, *t*_(17)_ = 0.596, *P* = 0.559; total distance traveled, *t*_(17)_ = 0.654, *P* *=* 0.522; female mice: time in the central zone, *t*_(16)_ = 1.110, *P* = 0.284; total distance traveled, *t*_(16)_ = 0.225, *P* = 0.825).

### Alterations in expression of key components of the 5-HT system induced by deletion of AdipoR1 in 5-HT neurons in both male and female mice

A functional 5-HT system in the brain requires endogenous 5-HT synthesis by TPH2 [58, 59] and reuptake of 5-HT by SERT [60], which constitute two essential functions of mature 5-HT neurons for proper 5-HT neurotransmission. We therefore determined expression levels of TPH2, SERT as well as 5-HT in the DRN and two projection fields of raphe 5-HT neurons.

**Fig. 4:**
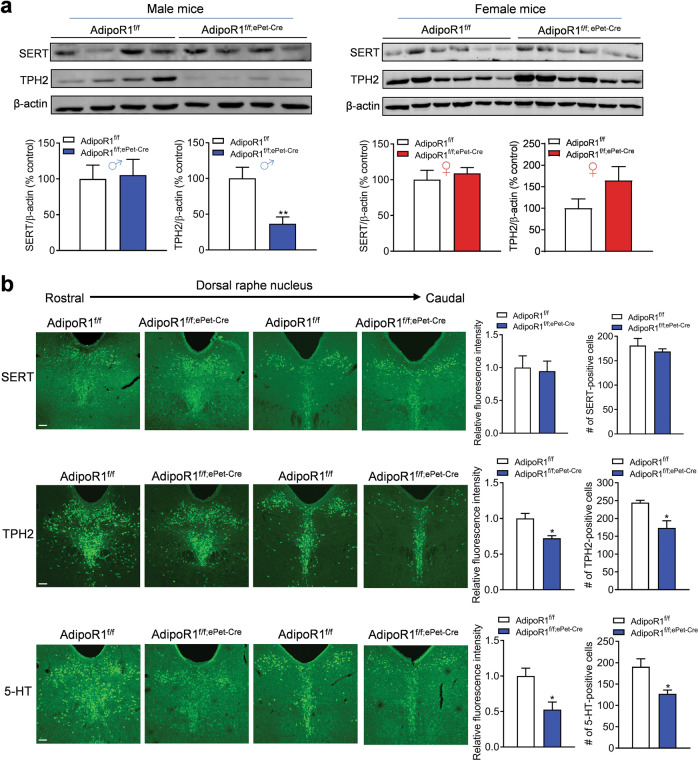
Effects of selective deletion of AdipoR1 in 5-HT neurons on expression levels of SERT, TPH2 and 5-HT in the dorsal raphe nucleus. **a** Immunoblots showing SERT and TPH2 protein levels in male (left) and female (right) AdipoR1^f/f^ and AdipoR1^f/f;ePet-Cre^ mice. Male mice: AdipoR1^f/f^, *n* = 6; AdipoR1^f/f;ePet-Cre^, *n* = 6. Female mice: AdipoR1^f/f^, *n* = 6; AdipoR1^f/f;ePet-Cre^, *n* = 6. **b** (Left panels) Representative immunohistochemical staining showing SERT, TPH2, and 5-HT immunofluorescence in the dorsal raphe nucleus of male AdipoR1^f/f^ and AdipoR1^f/f;ePet-Cre^ mice. (Middle panels) Relative fluorescence intensity normalized by control mice. (Right panels) The average number of immunopositive neurons per section in the dorsal raphe nucleus. SERT: 12 sections from three AdipoR1^f/f^ mice; 12 sections from three AdipoR1^f/f;ePet-Cre^ mice. TPH2: 11 sections from three AdipoR1^f/f^ mice; 11 sections from three AdipoR1^f/f;ePet-Cre^ mice. 5-HT: 12 sections from three AdipoR1^f/f^ mice; 12 sections from three AdipoR1^f/f;ePet-Cre^ mice. Scale bar = 100 µm. *P < 0.05, **P < 0.01 compared with AdipoR1^f/f^ littermate controls.

In the DRN, loss of AdipoR1 in 5-HT neurons did not affect SERT protein levels in either male or female mice (Fig. 4a, male, *t*_(10)_ = 0.180, *P* = 0.861; female, *t*_(10)_ = 0.582, *P* = 0.573). Interestingly, TPH2 protein levels were decreased in male AdipoR1^f/f;ePet-Cre^ mice (Fig. 4a, *t*_(10)_ = 3.487, *P* = 0.006), but not significantly altered in female AdipoR1^f/f;ePet-Cre^ mice (*t*_(10)_ = 1.654, *P* = 0.129) when compared with AdipoR1^f/f^ mice. We further analyzed SERT, TPH2, and 5-HT immunoreactivity in male mice. The number of SERT-immunopositive cells and total fluorescence intensity in the DRN showed no genotype differences (Fig. 4b, fluorescence intensity: *t*_(4)_ = 0.234, *P* = 0.827; cell number: *t*_(4)_ = 0.773, *P* = 0.483). By contrast, the numbers of TPH2- and 5-HT-immunopositive cells and fluorescence intensity of TPH2 and 5-HT were significantly decreased by the deletion of AdipoR1 in 5-HT neurons (Fig. 4b; TPH2: fluorescence intensity: *t*_(4)_ = 3.578, *P* = 0.023; cell number: *t*_(4)_ = 3.367, *P* = 0.028; 5-HT: fluorescence intensity: *t*_(4)_ = 3.102, *P* = 0.036; cell number: *t*_(4)_ = 3.061, *P* = 0.038).

**Fig. 5:**
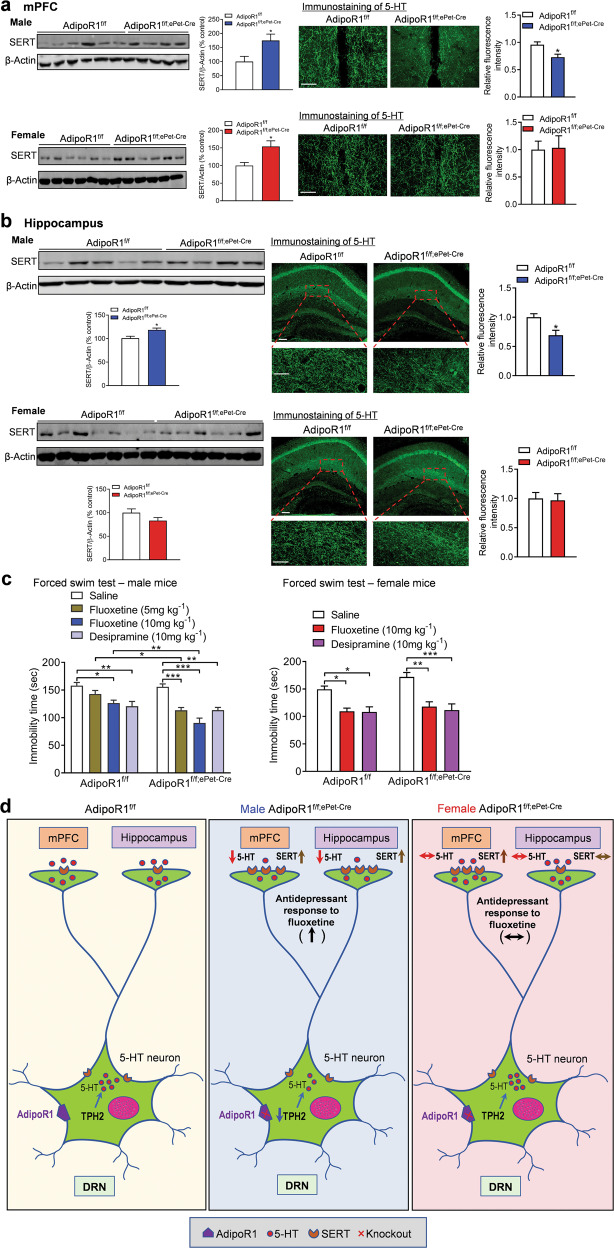
Effects of selective deletion of AdipoR1 in 5-HT neurons on SERT and 5-HT immunoreactivity in mPFC and hippocampus and behavioral responses to fluoxetine and desipramine. **a** (Left panels) Representative immunoblots and quantitative analysis of SERT levels in the mPFC of male (upper) and female (lower) mice. Male mice: AdipoR1^f/f^, *n* = 6; AdipoR1^f/f;ePet-Cre^, *n* = 6. Female mice: AdipoR1^f/f^, *n* = 7. AdipoR1^f/f;ePet-Cre^, *n* = 7. (Right panels) Representative brain sections immunostained for 5-HT and quantitative analysis of the intensity of 5-HT-positive fibers in the mPFC of male (upper) and female (lower) mice. Male: 14 sections from three AdipoR1^f/f^ mice; 14 sections from three AdipoR1^f/f;ePet-Cre^ mice. Female: 14 sections from three AdipoR1^f/f^ mice; 13 sections from three AdipoR1^f/f;ePet-Cre^ mice. Scale bar = 50 µm. **b** (Left panels) Representative immunoblots and quantitative analysis of SERT levels in the hippocampus of male (upper) and female (lower) mice. Male mice: AdipoR1^f/f^, *n* = 6; AdipoR1^f/f;ePet-Cre^, *n* = 6. Female mice: AdipoR1^f/f^, *n* = 7. AdipoR1^f/f;ePet-Cre^, *n* = 7. (Right panels) Representative brain sections immunostained for 5-HT and quantitative analysis of the intensity of 5-HT-positive fibers in the hippocampus of both male (upper) and female (lower) mice. Male: 24 sections from three AdipoR1^f/f^ mice; 23 sections from three AdipoR1^f/f;ePet-Cre^ mice. Female: 21 sections from three AdipoR1^f/f^ mice; 24 sections from three AdipoR1^f/f;ePet-Cre^ mice. Scale bar = 100 µm for low magnification and 50 µm for high magnification. **c** Forced swim test performed 30 min after fluoxetine (5 or 10 mg kg^−1^, i.p.) or desipramine (10 mg kg^−1^, i.p.) injection in male (left) and female (right) mice. Male mice: AdipoR1^f/f^ + saline, *n* = 7; AdipoR1^f/f^ + fluoxetine (5 mg kg^−1^), *n* = 9; AdipoR1^f/f^ + fluoxetine (10 mg kg^−1^), *n* = 7; AdipoR1^f/f^ + desipramine, *n* = 9; AdipoR1^f/f;ePet-Cre^ + saline, *n* = 7; AdipoR1^f/f;ePet-Cre^ + fluoxetine (5 mg kg^−1^), *n* = 9; AdipoR1^f/f;ePet-Cre^ + fluoxetine (10 mg kg^−1^), *n* = 8; AdipoR1^f/f;ePet-Cre^ + desipramine, *n* = 7. Female mice: AdipoR1^f/f^ + saline, *n* = 8; AdipoR1^f/f^ + fluoxetine, *n* = 7; AdipoR1^f/f^ + desipramine, *n* = 10; AdipoR1^f/f;ePet-Cre^ + saline, *n* = 7; AdipoR1^f/f;ePet-Cre^ + fluoxetine, *n* = 7; AdipoR1^f/f;ePet-Cre^ + desipramine, *n* = 10. *P < 0.05, **P < 0.01, ***P < 0.001 compared with AdipoR1^f/f^ littermate controls or saline-treated groups. **d** Schematic diagram illustrating the alterations in 5-HT system components and antidepressant responses induced by the loss of AdipoR1 in 5-HT neurons.

In the two DRN projection fields, i.e., hippocampus and mPFC, SERT protein expression and 5-HT immunoreactivity were examined. Protein levels of SERT were increased in both the mPFC and hippocampus of male AdipoR1^f/f;ePet-Cre^ mice (Fig. 5a, b; mPFC: *t*_(10)_ = 2.549, *P* *=* 0.029; hippocampus: *t*_(10)_ = 2.969, *P* = 0.014) but only in the mPFC of female AdipoR1^f/f;ePet-Cre^ mice when compared with AdipoR1^f/f^ mice (Fig. 5a, b; mPFC: *t*_(12)_ = 2.912, *P* = 0.013; hippocampus: SERT: *t*_(12)_ = 1.616, *P* = 0.132). In consistence with the reduced TPH2 and 5-HT levels in the DRN, 5-HT immunofluorescence intensity was significantly decreased in the mPFC and hippocampus of male AdipoR1^f/f;ePet-Cre^ mice (Fig. 5a, b; mPFC: *t*_(4)_ = 3.072, *P* = 0.037; hippocampus: *t*_(4)_ = 2.966, *P* = 0.041), while female AdipoR1^f/f;ePet-Cre^ mice showed no change (Fig. 5a, b; mPFC: *t*_(4)_ = 0.127, *P* = 0.905; hippocampus: *t*_(4)_ = 0.207, *P* = 0.847), compared with AdipoR1^f/f^ mice.

### Loss of AdipoR1 signaling in 5-HT neurons enhances the antidepressant response to the SSRI fluoxetine

Given the alterations in SERT and TPH2 in the DRN and its projection fields, we next examined the effects of AdipoR1 deletion in 5-HT neurons on antidepressant-like behavioral responses to fluoxetine, a SSRI, in comparison with desipramine, a potent inhibitor of the reuptake of norepinephrine, with little effect on 5-HT reuptake [29, 61]. In the forced swim test, two-way ANOVA revealed significant effects of genotype (*F*_(1,55)_ = 15.830, *P* < 0.001), treatment (*F*_(3,55)_ = 18.590, *P* < 0.001) and genotype × treatment interaction (*F*_(3,55)_ = 3.020, *P* = 0.037) on immobility time in male mice. A lower dose of fluoxetine (5 mg kg^−1^) failed to alter immobility time in male AdipoR1^f/f^ control mice (*P* = 0.738), but significantly decreased immobility time in male AdipoR1^f/f;ePet-Cre^ mice (*P* < 0.001). A higher dose of fluoxetine (10 mg kg^−1^) and desipramine (10 mg kg^−1^) significantly reduced immobility time in both male AdipoR1^f/f^ and AdipoR1^f/f;ePet-Cre^ mice (Fluoxetine—10 mg kg^−1^: AdipoR1^f/f^, *P* = 0.048; AdipoR1^f/f;ePet-Cre^, *P* < 0.001. Desipramine—10 mg kg^−1^: AdipoR1^f/f^, *P* = 0.005; AdipoR1^f/f;ePet-Cre^, *P* = 0.002). Male AdipoR1^f/f;ePet-Cre^ mice displayed an enhanced behavioral response to fluoxetine (Fluoxetine—5 mg kg^−1^: *P* = 0.028; 10 mg kg^−1^: *P* = 0.009) but not to desipramine (*P* = 0.994). Female mice showed significant effects of treatment (*F*_(2,43)_ = 18.950, *P* < 0.001) but not genotype (*F*_(1,43)_ = 2.361, *P* = 0.132) and genotype × treatment interaction (*F*_(2,43)_ = 0.601, *P* = 0.553) on immobility time in the forced swim test. Fluoxetine and desipramine decreased immobility time in both female AdipoR1^f/f^ control mice and AdipoR1^f/f;ePet-Cre^ mice (Fluoxetine—10 mg kg^−1^: AdipoR1^f/f^, *P* = 0.048; AdipoR1^f/f;ePet-Cre^, *P* = 0.004. Desipramine—10 mg kg^−1^: AdipoR1^f/f^, *P* = 0.019; AdipoR1^f/f;ePet-Cre^, *P* < 0.001). However, there was no genotype difference in either fluoxetine (*P* = 0.989) or desipramine treatment (*P* = 0.999) (Fig. 5c).

## Discussion

We have previously shown that adiponectin haploinsufficiency causes depression- and anxiety-related behaviors in mice via a central mechanism of action [21, 24]. The present study provides evidence of 5-HT neurons as a neuronal substrate mediating the effects of adiponectin/AdipoR1 signaling on depression- but not anxiety-related behaviors. One important finding was that 5-HT neuron-specific AdipoR1 knockout causes differential behavioral phenotypes, stress susceptibility and antidepressant responses in male and female mice. The underlying mechanisms seem to involve alterations in the key components of 5-HT transmission. We demonstrated that AdipoR1 is required for normal expression of genes involved in synthesis and reuptake of 5-HT.

It is well recognized that gender differences in depressive symptoms and antidepressant response, the neurobiological basis, however, remains poorly understood. Depressive disorders are more prevalent in people with obesity and diabetes [62–66]. Circulating levels of adiponectin are decreased in obesity and type 2 diabetes [67] and are lower in men than in women [47, 68]. We have previously shown that social stress reduces plasma adiponectin levels and adiponectin haploinsufficiency increases stress susceptibility in male mice [40]. Female mice appear to be more resilient to stress-induced reduction of adiponectin (data not shown). We speculate that sex differences may exist in the sensitivity of adiponectin receptors expressed in specific neuronal populations. Adiponectin AdipoR1 and AdipoR2 receptors have distinct distribution patterns [21]. While AdipoR1 is widely distributed in the brain, expression of AdipoR2 is restricted to only few brain regions [21]. Our in situ hybridization data revealed that AdipoR1 is the predominant receptor that mediates adiponectin actions in the DRN. Furthermore, we demonstrated that AdipoR1 was expressed in the vast majority of 5-HT neurons, suggesting a direct action of adiponectin on DRN 5-HT neurons through AdipoR1. To assess the specific role of AdipoR1 in 5-HT neurons, we used an ePet-Cre transgenic mouse line to delete floxed AdipoR1 specifically in 5-HT neurons [45]. Pet-1 is a precise marker of developing and adult 5-HT neurons [46, 69, 70]. We confirmed that Cre activity in ePet-Cre mice was restricted to 5-HT neurons defined by TPH2 and SERT immunoreactivity. This cell specificity of ePet-Cre transgenic line is an advantage over the SERT-Cre knockin mouse line, in which Cre recombinase is much more widely expressed in the brain and periphery [71]. We found that male mice lacking AdipoR1 in 5-HT neurons displayed reduced sex-related reward-seeking behavior, assessed by the female urine sniffing test. While both male and female conditional knockout mice showed no change in hedonic responses to sucrose under basal conditions, a reduction in the preference for saccharin, a non-caloric sweetener, was observed in male but not female mice. Interestingly, loss of AdipoR1 in 5-HT neurons enhanced stress-induced decrease in sucrose preference in both sexes. The caloric content in sucrose solution and limited palatability of saccharin compared with sucrose may account for differences in the sensitivity to sucrose and saccharin [72]. In addition, female but not male conditional knockout mice exhibited enhanced behavioral despair in the forced swim test. These differences in phenotypic expression between male and female mice are unlikely due to the different degree of AdipoR1 expression in 5-HT neurons or AdipoR1 deletion induced by ePet-Cre as male and female mice have similar AdipoR1 colocalization patterns and comparable extents of AdipoR1 deletion in the DRN.

The mechanisms by which AdipoR1 in 5-HT neurons regulates depression-related behaviors appear to involve regulation of two key components of 5-HT homeostasis, i.e., synthesis and reuptake. 5-HT synthesis is controlled by two isoforms of TPH, TPH1 and TPH2, which catalyzes the rate-limiting reaction in the biosynthesis of 5-HT in the periphery and brain, respectively [58, 73]. TPH2 is predominantly expressed in 5-HT neurons of the raphe nuclei and converts L-tryptophan into L-5-hydroxytryptophan, a precursor for brain 5-HT synthesis [58, 74]. The role of 5-HT synthesis in the pathophysiology of depression is suggested by the fact that acute tryptophan depletion can induce a depressive state in patients with remitted depression [75]. Furthermore, the findings of low 5-HT and its metabolite 5-HIAA detected in the CSF of suicides and depressed patients indicate a state of brain 5*-*HT deficiency [76]. Consistently, loss-of-function mutations of TPH2 have been associated with susceptibility to major depression and suicidal behavior [36, 77–80]. It has been reported that the mean rate of 5-HT synthesis in normal males is 52% higher than in normal females [81], but the rate of brain serotonin metabolism is higher in females than in males [82, 83]. We found that selective deletion of AdipoR1 in 5-HT neurons decreased TPH2 levels in the DRN with concurrent reduction of 5-HT immunoreactivity at the cell body region and two terminal areas, i.e., the mPFC and hippocampus, of male but not female mice. The reduced 5-HT synthesis would result in decreased 5-HT release and transmission. Once released from nerve terminals, the action of 5-HT in the synaptic cleft is terminated primarily by reuptake into presynaptic nerve terminals by SERT that can be inhibited by fluoxetine. While loss of AdipoR1 in 5-HT neurons had no effects on SERT protein expression in the DRN, it increased SERT protein levels in both the mPFC and hippocampus of male mice, but only in the mPFC of female mice. Decreased 5-HT synthesis and/or increased reuptake in male mice presumably lead to deficient 5-HT neurotransmission to different extents in male and female mice. The greater behavioral deficits in male mice may result from the combined effects of decreased synthesis and increased uptake of 5-HT in both the mPFC and hippocampus, while the modest overt phenotypes observed in female mice may be due to increased SERT expression in the mPFC. In consistence with robust increases in SERT levels in multiple brain regions, male mice lacking AdipoR1 in 5-HT neurons showed enhanced antidepressant-like behavioral responses to the serotonin reuptake inhibitor fluoxetine. These results suggest that AdipoR1 receptors are required for regulation of normal 5-HT neurotransmission and dysfunctional AdipoR1 signaling causes depression-related behaviors that are highly responsive to SERT inhibition.

The common behavioral phenotype observed in both male and female mice lacking AdipoR1 in 5-HT neurons is anhedonia. Previous studies have suggested that 5-HT neurons in the DRN encode reward signals [50, 51, 84, 85]. Rewards activate 5-HT neurons [51], and stimulation of 5-HT neurons induces self-administration [50] and resembles the natural reward of sucrose [84]. Our data suggest that adiponectin/AdipoR1 signaling plays an important role in reward processing of 5-HT neurons under basal and stress conditions.

In conclusion, our results demonstrate an important role of adiponectin/AdipoR1 signaling in 5-HT neurons in depression-related behaviors and antidepressant responses, which involves in the regulation of key components of 5-HT neurotransmission. These effects are more pronounced in males than females. Our observations provide a basis for a novel mechanism by which dysfunctional adipose tissue-brain crosstalk contributes to mood regulation. Investigations of how AdipoR1 signaling influences 5-HT neuron activity and transmission will reveal new insights into our understanding of mood disorders, especially those conditions that are comorbid with obesity and type 2 diabetes involving adipose tissue dysfunction and hypoadiponectinaemia.
